# Supplementary figures and images for: Disentangling group specific QTL allele effects from genetic background epistasis using admixed individuals in GWAS: An application to maize flowering
Source: PLoS Genet. 2020 Mar 4;16(3):e1008241. doi: 10.1371/journal.pgen.1008241 (PMC7075643; doi:10.1371/journal.pgen.1008241)

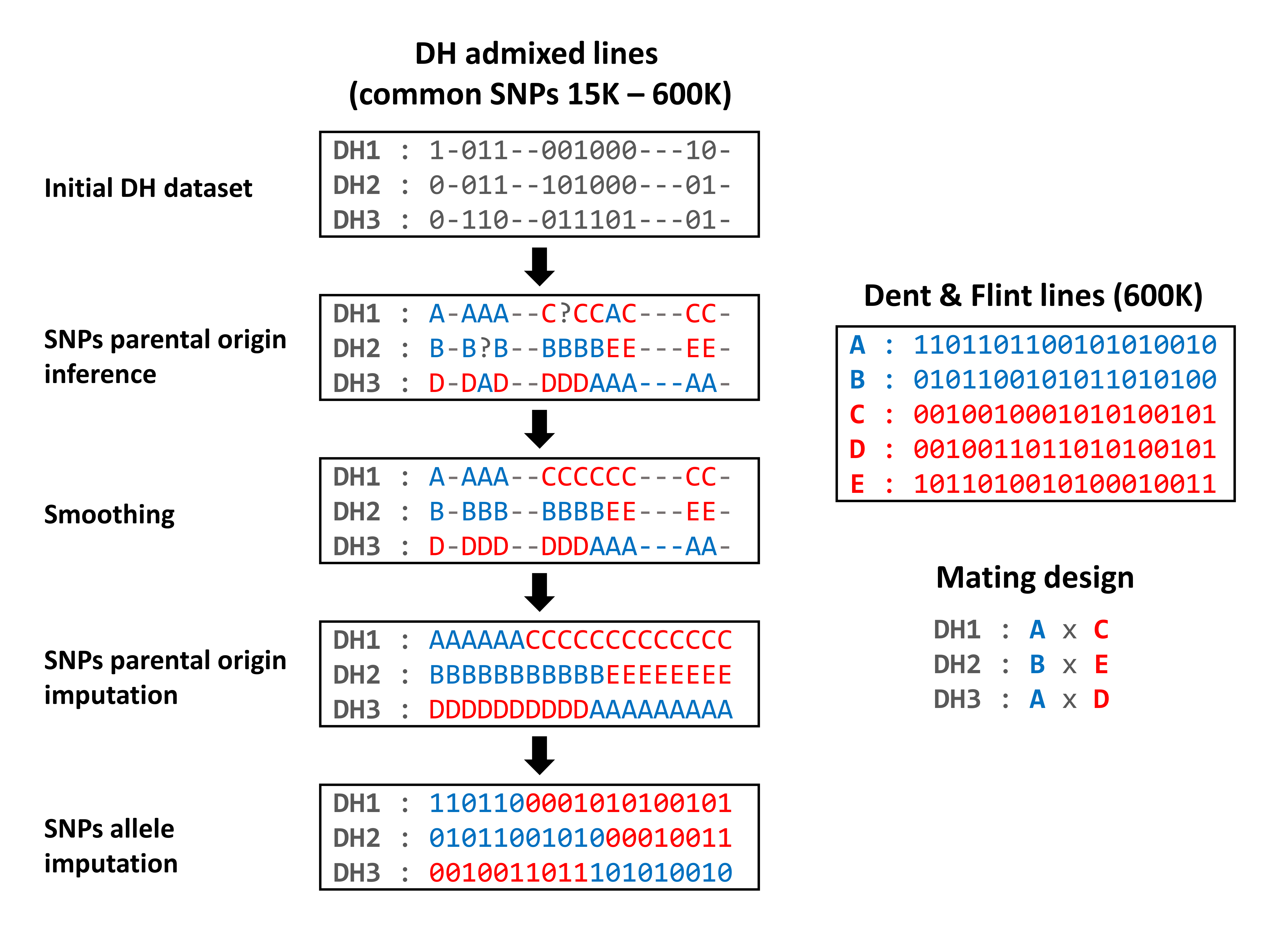

Supplement: S1 Fig — Diagram illustrating the procedure applied to impute admixed DH lines from 15K to 600K SNPs using the parental origin of alleles. (TIF) [file pgen.1008241.s001.tif]

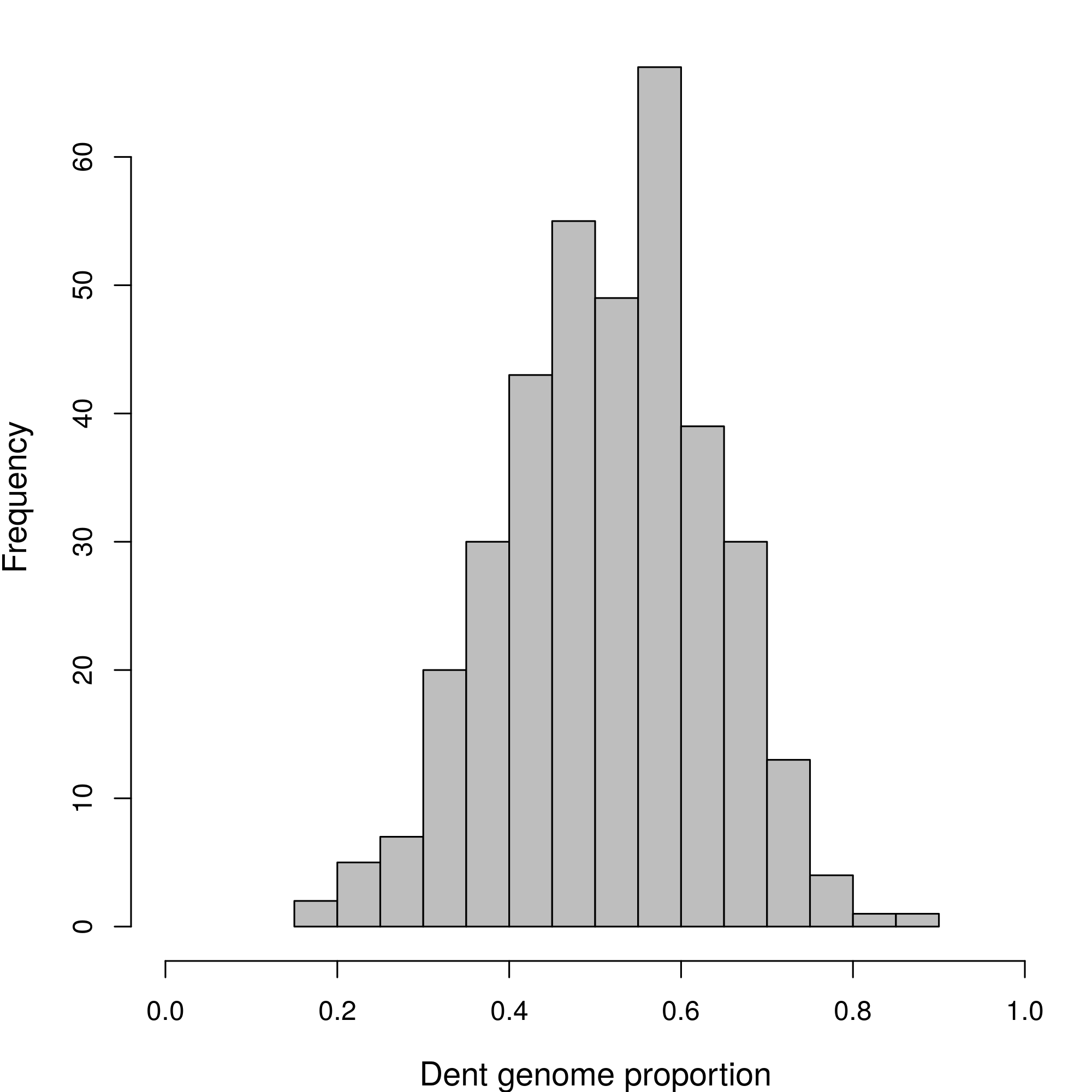

Supplement: S2 Fig — (TIF) [file pgen.1008241.s002.tif]

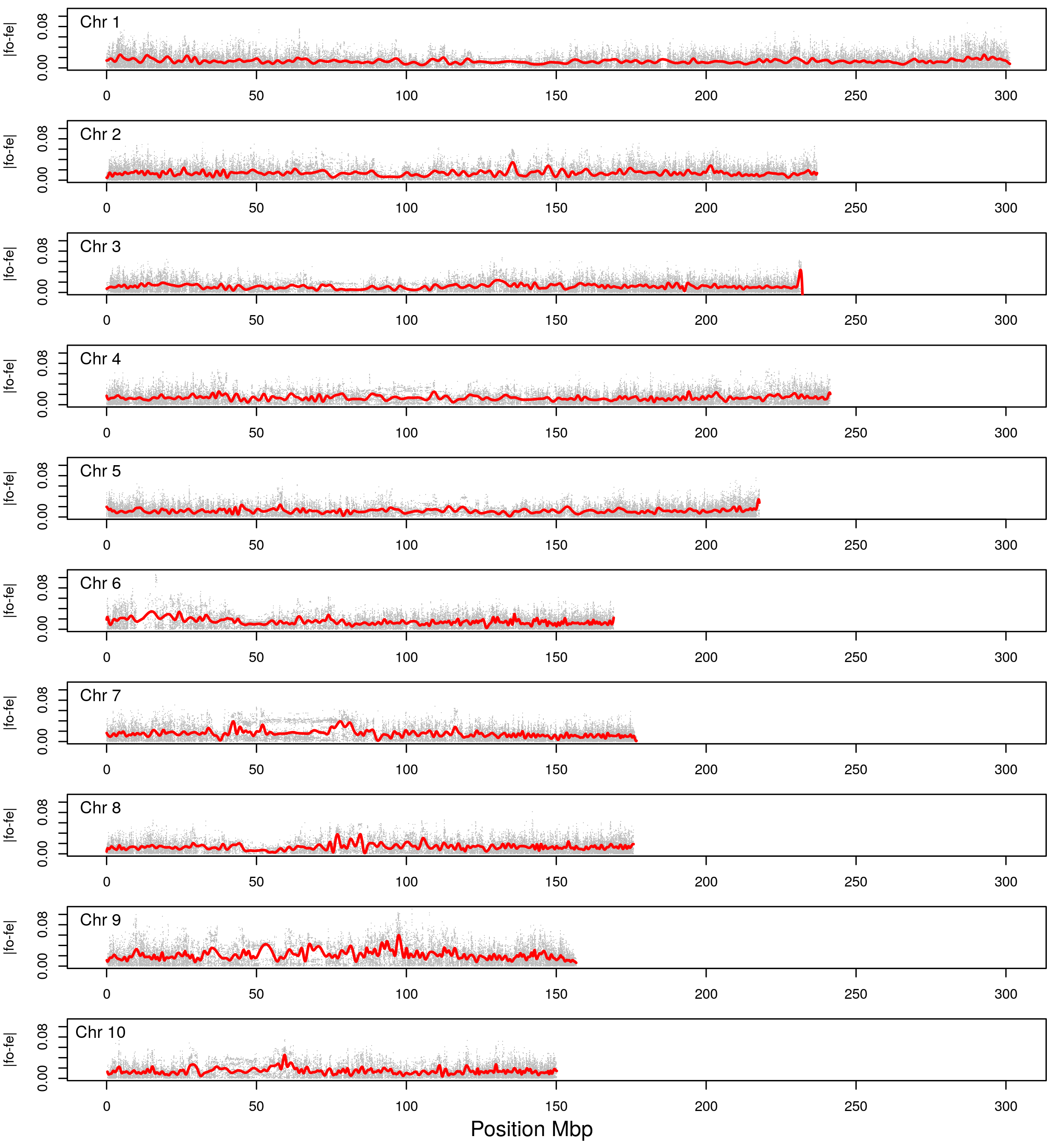

Supplement: S3 Fig — Absolute difference between observed allele frequency of the reference allele fo estimated on the admixed lines and their expected value fe along each chromosome (|fo − fe|). The expected allele frequencies were computed as the mean of flint and dent allele frequencies estimated on the parental lines by taking into account the contribution of each parent. A cubic smoothing spline was adjusted using the R function “smooth.spline”, and plotted in red. (TIF) [file pgen.1008241.s003.tif]

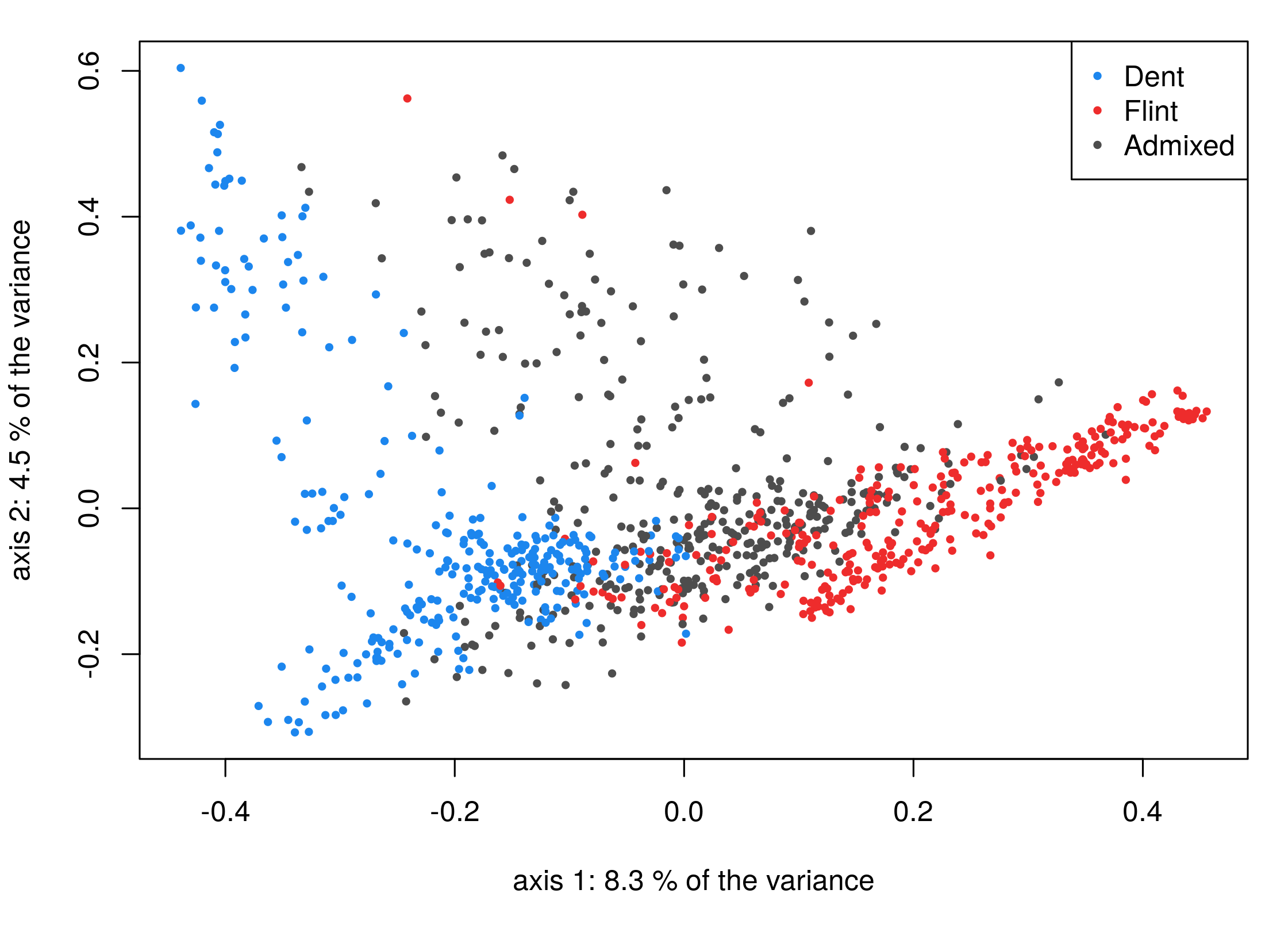

Supplement: S4 Fig — Individuals were colored depending on their genetic background: dent, flint or admixed. (TIF) [file pgen.1008241.s004.tif]

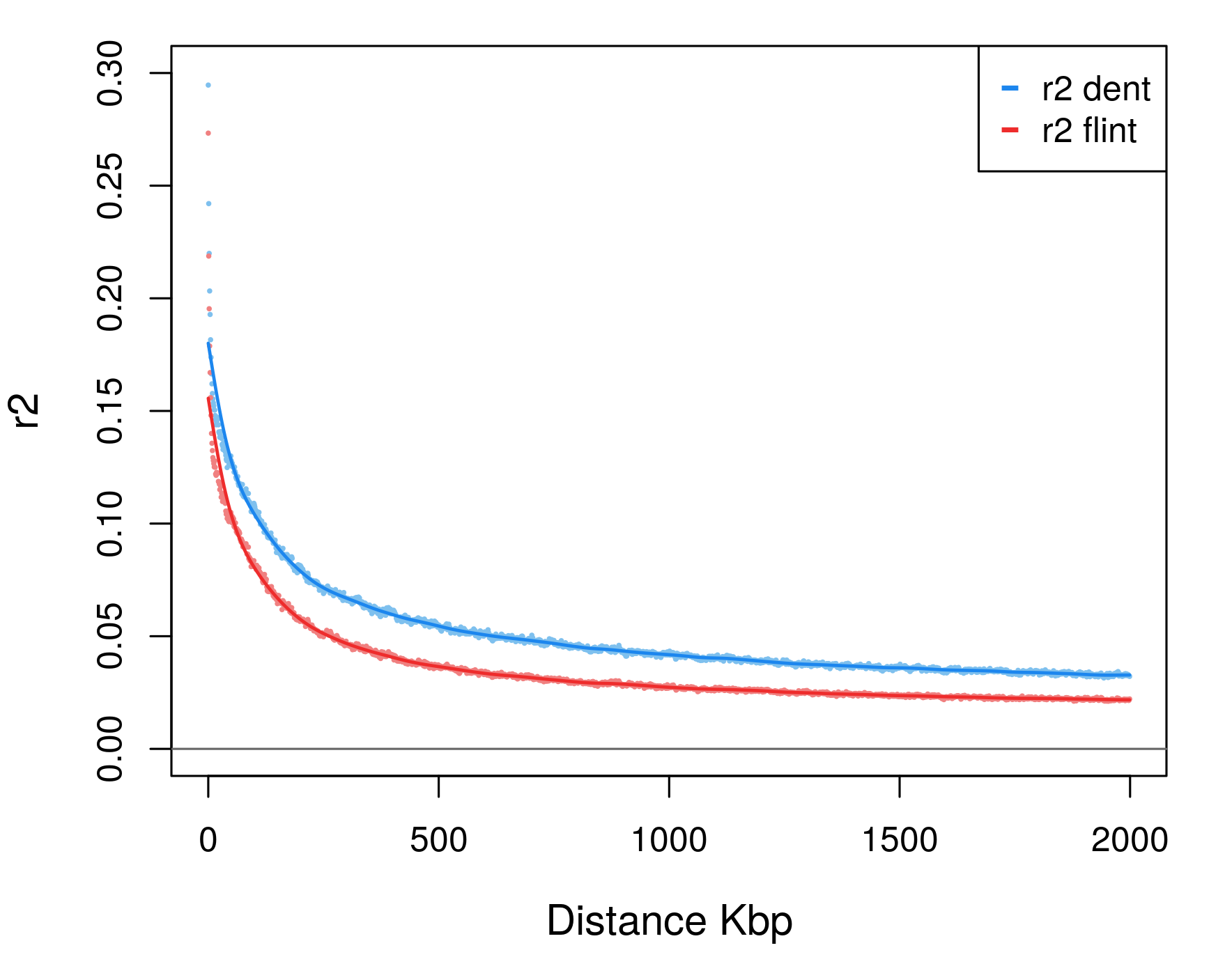

Supplement: S5 Fig — LD extent estimated separately in dent and flint genetic groups using the standard r2. LD was calculated and averaged for loci pairs characterized by a similar physical distance ranging from 0 to 2 Mbp, considering a sliding window of 1Kbp. A cubic smooth spline was adjusted for each group, using the R function “smooth.spline”. (TIF) [file pgen.1008241.s005.tif]

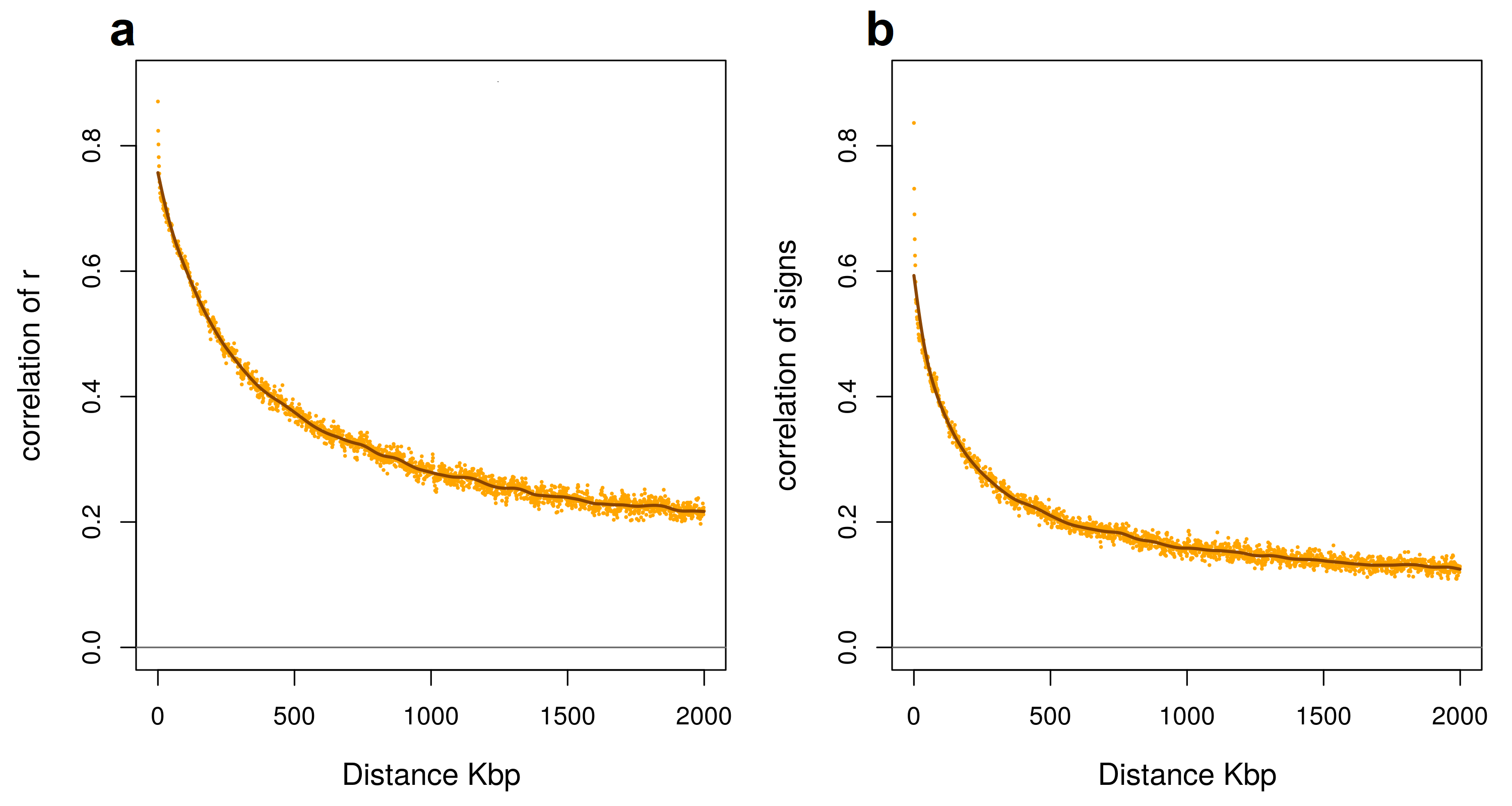

Supplement: S6 Fig — Conservation of LD phases estimated using the correlation (a) between the r of dent and flint groups, and (b) between the signs of r in the dent and flint groups. LD was calculated and averaged for loci pairs characterized by a similar physical distance ranging from 0 to 2 Mbp, considering a sliding window of 1Kbp. A cubic smooth spline was adjusted for each method, using the R function “smooth.spline”. (TIF) [file pgen.1008241.s006.tif]

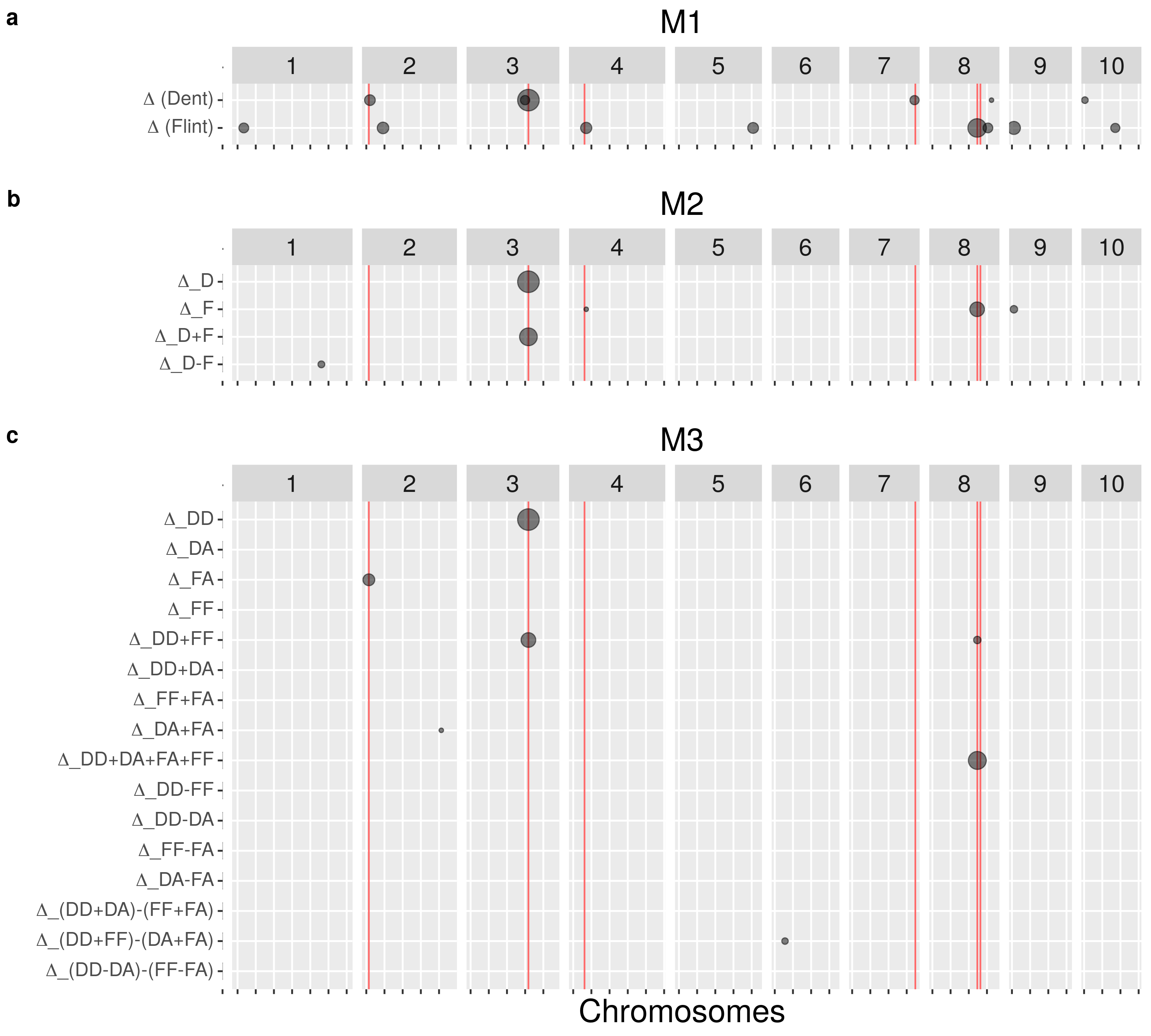

Supplement: S7 Fig — Position of QTLs detected for FF with a FDR of 20% using (a) M1, (b) M2 and (c) M3. The size of the grey dots is proportional to the -log10(pval) of the test at the most significant SNP of the region. (TIF) [file pgen.1008241.s007.tif]

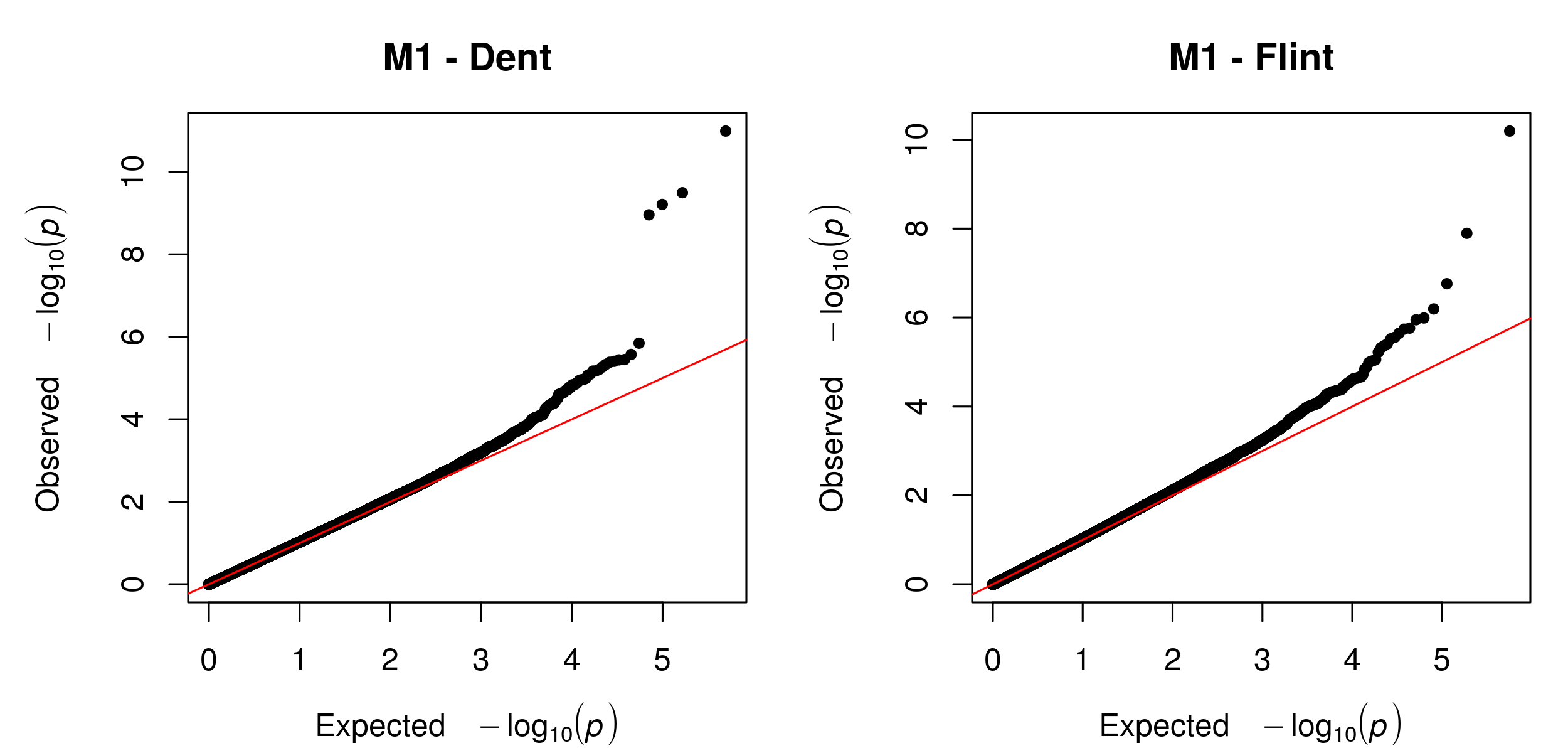

Supplement: S8 Fig — (TIF) [file pgen.1008241.s008.tif]

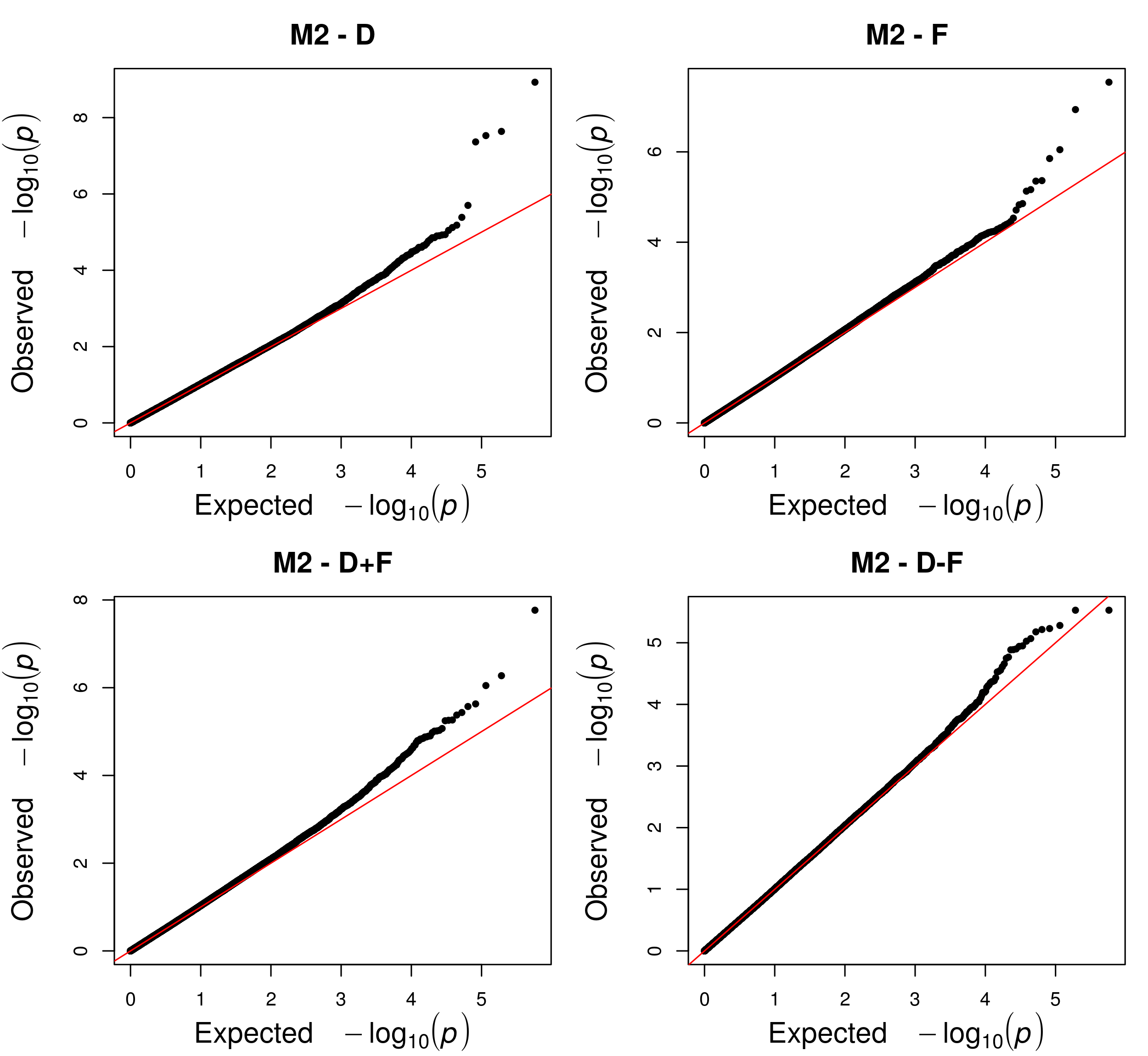

Supplement: S9 Fig — (TIF) [file pgen.1008241.s009.tif]

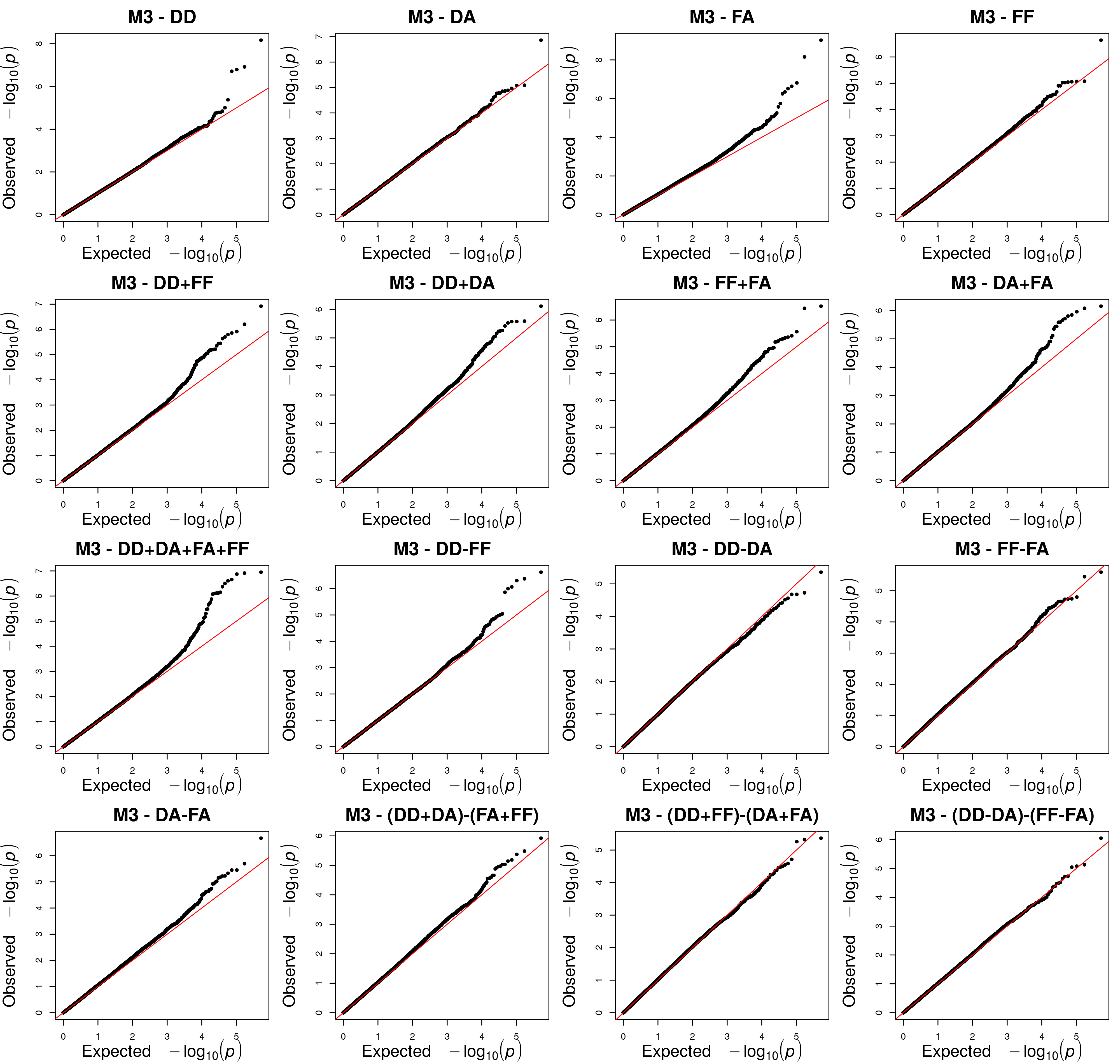

Supplement: S10 Fig — (TIF) [file pgen.1008241.s010.tif]

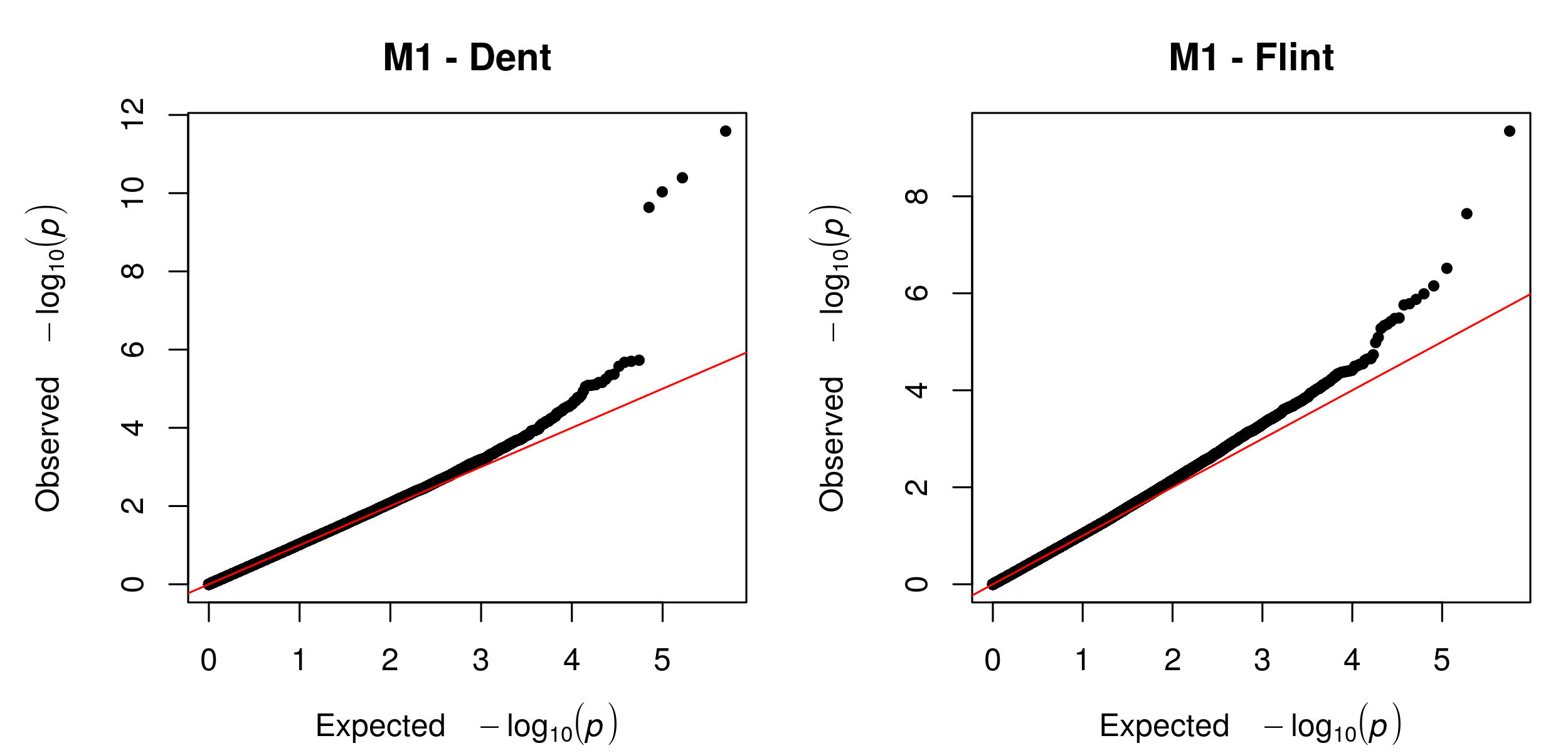

Supplement: S11 Fig — (TIF) [file pgen.1008241.s011.tif]

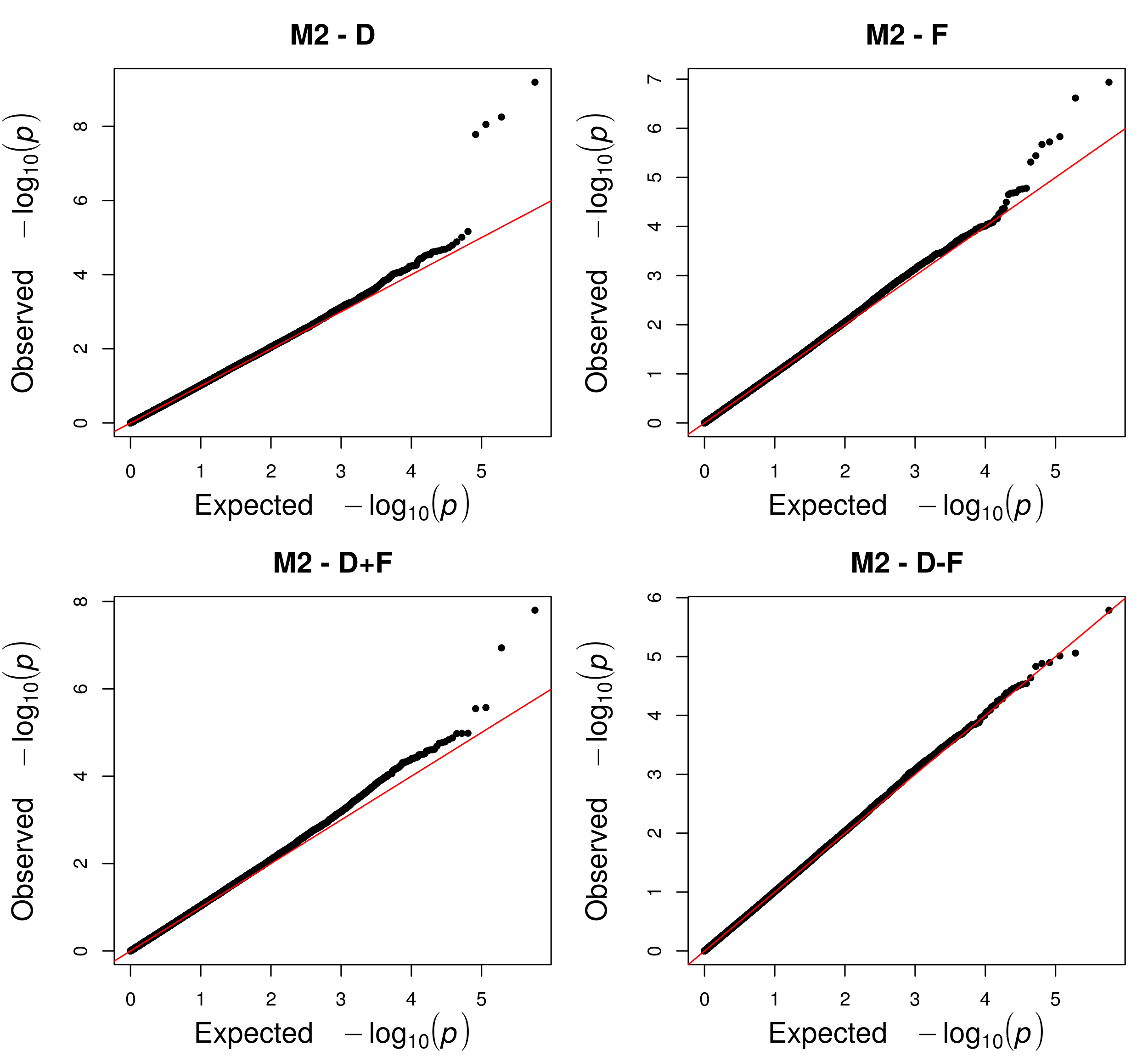

Supplement: S12 Fig — (TIF) [file pgen.1008241.s012.tif]

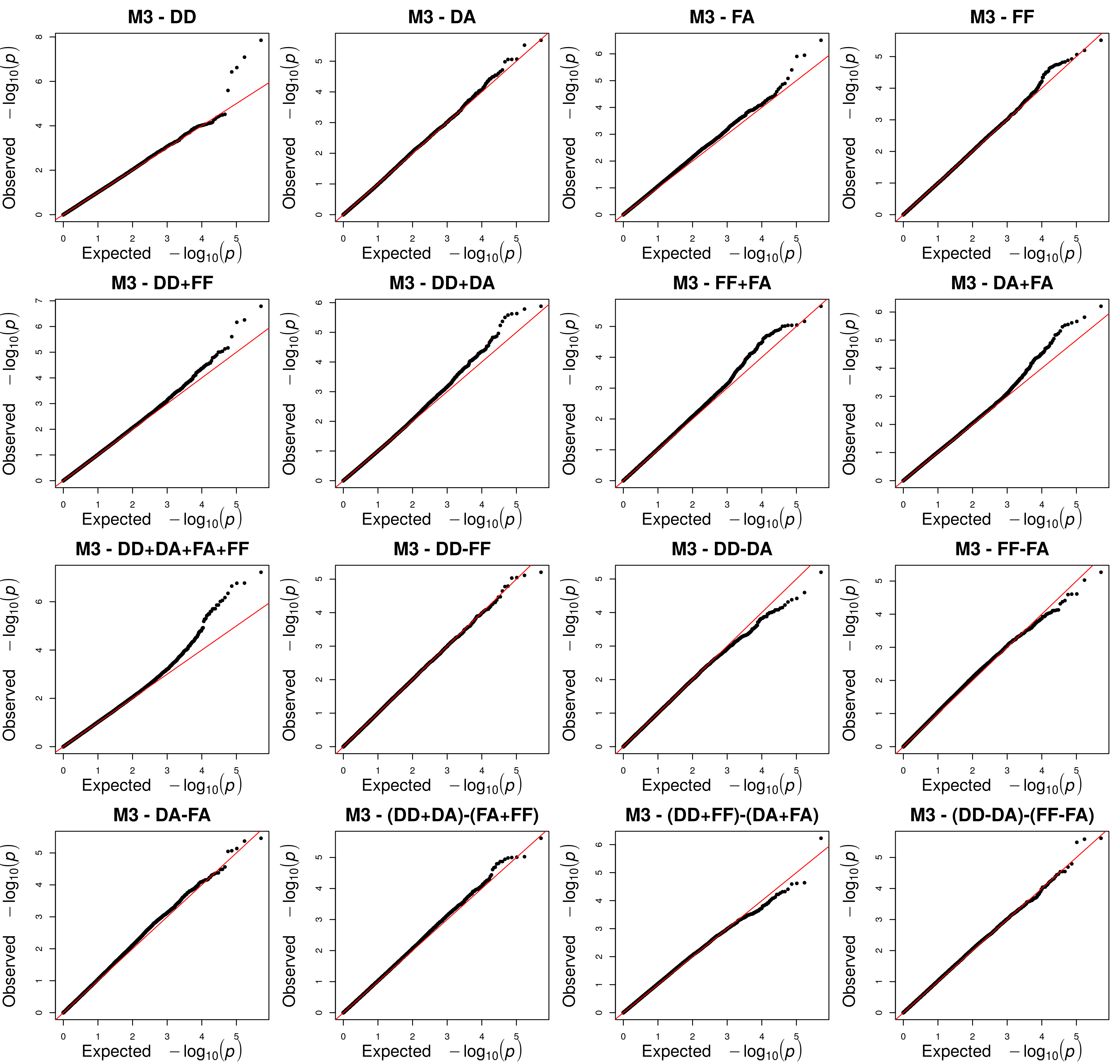

Supplement: S13 Fig — (TIF) [file pgen.1008241.s013.tif]
