# Supplementary material for: Disentangling group specific QTL allele effects from genetic background epistasis using admixed individuals in GWAS: An application to maize flowering
Source: PLoS Genet. 2020 Mar 4;16(3):e1008241. doi: 10.1371/journal.pgen.1008241 (PMC7075643; doi:10.1371/journal.pgen.1008241)
Supplement: S2 Appendix — In this appendix, the properties of the new GWAS models were evaluated in terms of false discovery rate and statistical power of the tests. (PDF) [file pgen.1008241.s020.pdf]

S2 Appendix - False discovery rate and statistical power of GWAS models

The false discovery rate and the statistical power of the GWAS models $\mathbf{M_1}$, $\mathbf{M_2}$ and $\mathbf{M_3}$ were investigated using simulations. The simulations were based on real genotypic data and simulated phenotypes.

Simulated phenotypes

One hundred genetic architectures were simulated using 16 QTLs sampled among 187,489 SNPs. The 187,489 SNPs are those selected after filtering on allele frequencies for both $\mathbf{M_1}$ (Dent), $\mathbf{M_1}$ (Flint) $\mathbf{M_2}$ and $\mathbf{M_3}$. For each architecture, a given chromosome remained free of QTLs and was called chromosome H_0. Each chromosome was the chromosome H_0 10 times among the 100 replicates. For a given genetic architecture, each of the 16 QTLs matched one of the 16 configurations of effects presented in the Table 3 of the article (e.g. Δ^m_{DD} corresponds to an effect associated with dent alleles in the dent genetic background only). A value of 1 was assigned to the difference between the effects of allele "1" and "0" for a given ancestry and genetic background (e.g. $\Delta^m_{DD} = \beta^m_{1DD} - \beta^m_{0DD} = 1$). For divergent allele effects between ancestries or genetic backgrounds, opposite signs were assigned to the different deltas (e.g. $\Delta^m_{DD-DA} = \Delta^m_{DD} - \Delta^m_{DA}$ with $\Delta^m_{DD} = 1$ and $\Delta^m_{DA} = -1$) . Genetic values were computed by summing the effects associated with the alleles carried by each individual at the 16 QTLs, while respecting their specificity in terms of ancestry or genetic background. To simulate phenotypes with 3 levels of heritability ($h^2 = \{0.2, 0.5, 0.8\}$), an environmental error was added to each individual genetic value by sampling in a normal distribution $N(0, \sigma^2_E)$ with $\sigma^2_E = \dfrac{(1-h^2)\sigma^2_G}{h^2}$ where σ^2_G is the sample variance of the simulated genetic values.

GWAS

The GWAS strategies $\mathbf{M_1}$ (Dent), $\mathbf{M_1}$ (Flint), $\mathbf{M_2}$ and $\mathbf{M_3}$ were applied to the 100 genetic architectures and 3 levels of heritability, using the settings presented in the article.

False discovery rate

The false discovery rate was investigated using chromosomes H_0, for which all tested hypotheses at SNPs are under H_0. The mean proportion of p-values below 1% and 1‰ was close to 1% and 1‰ respectively for all GWAS models (Table A). For each GWAS model and genetic architecture, the observation of QQ-plots of the global set of p-values did not reveal any major issues in terms of expected behavior (Fig A, B, C and D). For $\mathbf{M_2}$ and $\mathbf{M_3}$, the QQ-plots corresponding to each type of test did not show any specific problem (Fig E, F, G, H, I and J). The FDR procedure described in the study was applied on chromosome H_0 using a nominal value of 5% or 20%. A realized FDR of 0 was obtained when no SNP was detected and a FDR of 1 was obtained when at least one SNP was detected on that chromosome. The realized FDRs were averaged over the 100 replicates and compared to the nominal value (5% or 20 %). For $\mathbf{M_2}$ and $\mathbf{M_3}$, the mean realized FDRs were either equal or below the nominal value. Considering $\mathbf{M_1}$ (Dent) and $\mathbf{M_1}$ (Flint), the mean realized FDRs

substantially exceeded the nominal value for a heritability of 0.2. In conclusion, the new GWAS models presented in the study (**M₂** and **M₃**) correctly control for false positives.

Statistical power

The statistical power of the tests of each GWAS model was investigated using the proportion of QTLs detected over the 100 genetic architectures for each level of heritability. A QTL was considered as detected when a SNP located at less than 1.5 Mbp from the simulated QTL was declared significant after controlling for multiple testing. The proportions are summarized in Tables C, D, E, F and G. The power of a given test depends on several factors: (i) the consistency between the QTL configuration of effect and the tested hypothesis (e.g. a QTL simulated according to Δ^m_{DD} could be detected using tests Δ^m (Dent), Δ^m_D or Δ^m_{DD} but not with tests Δ^m (Flint), Δ^m_F or Δ^m_{FF}), (ii) the number of observations involved in calculations (e.g. a higher power was observed for test $\Delta^m_{DD+DA+FA+FF}$ compared to tests Δ^m_{DD} and Δ^m_{DD+FF} when a QTL was simulated according to $\Delta^m_{DD+DA+FA+FF}$), (iii) the value of the simulated delta (e.g. a higher power was observed for test Δ^m_{DD-FF} when a QTL was simulated according to $\Delta^m_{DD-FF} = 2$ compared to a QTL simulated according to $\Delta^m_{DD} = 1$, even though both QTL configurations of effets are consistent with the tested hypothesis) and (iv) the level of heritability as a higher power was generally observed when $h^2 = 0.8$ compared to $h^2 = 0.2$. Note that non-null proportions could be observed for tests aiming at detecting QTLs that were not consistent with their configuration of effect, showing the existence of false positives (e.g. a non-null proportion for a QTL simulated according to Δ^m_{DD} that was detected using Δ^m_{FF}). However, such proportions were close to 0. In conclusion, the GWAS models presented in the study showed a good power for detecting QTLs that account for a substantial part of variance for traits with moderate to high heritabilities.

Tables

Table A. Mean proportion of p-values below 1% or 1‰ on chromosome H_0 over the 100 genetic architectures and 3 levels of heritability. The means are presented in 1% or 1‰

	$h^2 = 0.2$		$h^2 = 0.5$		$h^2 = 0.8$	
	1%	1‰	1%	1‰	1%	1‰
**M₁** (Dent)	1.08 (0.36)	1.18 (0.76)	1.12 (0.32)	0.97 (0.59)	0.87 (0.29)	0.90 (0.59)
**M₁** (Flint)	1.18 (0.36)	1.39 (0.77)	1.04 (0.32)	1.13 (0.59)	0.93 (0.26)	0.96 (0.54)
**M₂**	1.02 (0.22)	1.07 (0.45)	0.93 (0.22)	0.91 (0.37)	0.84 (0.20)	0.81 (0.37)
**M₃**	0.95 (0.21)	0.93 (0.39)	0.85 (0.18)	0.78 (0.26)	0.77 (0.17)	0.68 (0.25)

Standard deviations over the 100 genetic architectures are indicated between brackets

Table B. Mean proportion of false positives in % (realized FDR) when applying a 5% or 20% FDR procedure on chromosome H_0 (realized FDR is either 0 or 1) to declare a SNP significant as described in the study

	$h^2 = 0.2$		$h^2 = 0.5$		$h^2 = 0.8$	
FDR	5%	20%	5%	20%	5%	20%
$\mathbf{M_1}$ (Dent)	0.09	0.28	0.07	0.18	0.08	0.28
$\mathbf{M_1}$ (Flint)	0.14	0.35	0.04	0.20	0.05	0.25
$\mathbf{M_2}$	0.05	0.20	0.01	0.11	0.05	0.19
$\mathbf{M_3}$	0.01	0.17	0.00	0.09	0.02	0.11

Table C. Power of strategy $\mathbf{M_1}$ when applied with a nominal FDR level fixed at 20%. The power is computed as the proportions of detected QTLs (averaged over the 100 genetic architectures), displayed for each level of heritability and each group in columns

	$h^2 = 0.2$		$h^2 = 0.5$		$h^2 = 0.8$	
QTL configuration \ Test	Δ^m (Dent)	Δ^m (Flint)	Δ^m (Dent)	Δ^m (Flint)	Δ^m (Dent)	Δ^m (Flint)
Δ^m_{DD}	0.10	0.01	0.41	0.02	0.80	0.03
Δ^m_{DA}	0.02	0.00	0.04	0.01	0.04	0.04
Δ^m_{FA}	0.00	0.00	0.00	0.01	0.04	0.04
Δ^m_{FF}	0.00	0.00	0.02	0.28	0.08	0.82
Δ^m_{DD+FF}	0.05	0.03	0.31	0.32	0.77	0.73
Δ^m_{DD+DA}	0.01	0.01	0.27	0.01	0.77	0.06
Δ^m_{FF+FA}	0.01	0.05	0.05	0.31	0.04	0.80
Δ^m_{DA+FA}	0.00	0.03	0.04	0.02	0.06	0.04
$\Delta^m_{DD+DA+FA+FF}$	0.00	0.03	0.36	0.33	0.86	0.80
Δ^m_{DD-FF}	0.05	0.07	0.42	0.38	0.88	0.78
Δ^m_{DD-DA}	0.05	0.01	0.36	0.01	0.80	0.06
Δ^m_{FF-FA}	0.00	0.08	0.03	0.31	0.05	0.79
Δ^m_{DA-FA}	0.02	0.01	0.02	0.02	0.02	0.04
$\Delta^m_{(DD+DA)-(FF+FA)}$	0.01	0.04	0.28	0.33	0.81	0.71
$\Delta^m_{(DD+FF)-(DA+FA)}$	0.02	0.05	0.35	0.28	0.79	0.70
$\Delta^m_{(DD-DA)-(FF-FA)}$	0.09	0.02	0.36	0.36	0.75	0.80

Table D. Power of strategy $\mathbf{M_2}$ when applied with a nominal FDR level fixed at 20%. The power is computed as the proportions of detected QTLs (averaged over the 100 genetic architectures), displayed for each level of heritability and each of the four tests in columns

	$h^2 = 0.2$				$h^2 = 0.5$				$h^2 = 0.8$			
QTL configuration \Test	Δ^m_D	Δ^m_F	Δ^m_{D+F}	Δ^m_{D-F}	Δ^m_D	Δ^m_F	Δ^m_{D+F}	Δ^m_{D-F}	Δ^m_D	Δ^m_F	Δ^m_{D+F}	Δ^m_{D-F}
Δ^m_{DD}	0.02	0.00	0.01	0.02	0.35	0.02	0.15	0.15	0.73	0.03	0.45	0.44
Δ^m_{DA}	0.01	0.00	0.00	0.02	0.03	0.01	0.01	0.02	0.03	0.03	0.06	0.06
Δ^m_{FA}	0.00	0.00	0.00	0.00	0.00	0.00	0.01	0.00	0.03	0.05	0.06	0.05
Δ^m_{FF}	0.00	0.00	0.00	0.00	0.02	0.25	0.10	0.09	0.07	0.82	0.34	0.42
Δ^m_{DD+FF}	0.01	0.01	0.04	0.00	0.25	0.28	0.68	0.05	0.75	0.76	0.99	0.22
Δ^m_{DD+DA}	0.00	0.00	0.00	0.00	0.22	0.00	0.08	0.09	0.75	0.04	0.39	0.45
Δ^m_{FF+FA}	0.00	0.01	0.00	0.00	0.05	0.30	0.08	0.07	0.03	0.79	0.40	0.33
Δ^m_{DA+FA}	0.00	0.02	0.00	0.00	0.04	0.01	0.00	0.05	0.05	0.03	0.04	0.08
$\Delta^m_{DD+DA+FA+FF}$	0.00	0.02	0.04	0.01	0.31	0.35	0.71	0.07	0.83	0.81	0.97	0.24
Δ^m_{DD-FF}	0.01	0.02	0.00	0.06	0.36	0.39	0.06	0.74	0.83	0.81	0.25	0.99
Δ^m_{DD-DA}	0.04	0.00	0.01	0.00	0.33	0.01	0.18	0.13	0.79	0.04	0.49	0.49
Δ^m_{FF-FA}	0.00	0.00	0.00	0.00	0.03	0.31	0.12	0.08	0.04	0.81	0.37	0.46
Δ^m_{DA-FA}	0.00	0.00	0.00	0.00	0.01	0.01	0.00	0.00	0.00	0.04	0.02	0.04
$\Delta^m_{(DD+DA)-(FF+FA)}$	0.00	0.04	0.00	0.04	0.30	0.30	0.05	0.67	0.83	0.78	0.24	0.97
$\Delta^m_{(DD+FF)-(DA+FA)}$	0.01	0.01	0.06	0.01	0.28	0.28	0.74	0.03	0.79	0.72	0.97	0.23
$\Delta^m_{(DD-DA)-(FF-FA)}$	0.04	0.01	0.00	0.06	0.30	0.32	0.05	0.76	0.72	0.80	0.33	0.98

Table E. Power of strategy $\mathbf{M_3}$ when applied with a nominal FDR level fixed at 20%. The power is computed as the proportions of detected QTLs (averaged over the 100 genetic architectures with $h^2 = 0.2$), displayed for each of the sixteen tests in columns. The 16 tests are numbered as $\{t_1, ..., t_{16}\}$ and each of them matches one of the QTL configurations indicated in lines (e.g. t_1 corresponds to testing $\Delta^m_{DD} = 0$, but also to the QTL configuration Δ^m_{DD})

QTL configuration \Test	t_1	t_2	t_3	t_4	t_5	t_6	t_7	t_8	t_9	t_{10}	t_{11}	t_{12}	t_{13}	t_{14}	t_{15}	t_{16}
t_1 - Δ^m_{DD}	0.00	0.00	0.00	0.00	0.00	0.00	0.00	0.00	0.00	0.00	0.00	0.00	0.00	0.00	0.00	0.00
t_2 - Δ^m_{DA}	0.00	0.00	0.00	0.00	0.00	0.00	0.00	0.00	0.00	0.00	0.00	0.00	0.00	0.00	0.00	0.00
t_3 - Δ^m_{FA}	0.00	0.00	0.00	0.00	0.00	0.00	0.00	0.00	0.00	0.00	0.00	0.00	0.00	0.00	0.00	0.00
t_4 - Δ^m_{FF}	0.00	0.00	0.00	0.00	0.00	0.00	0.00	0.00	0.00	0.00	0.00	0.00	0.00	0.00	0.00	0.00
t_5 - Δ^m_{DD+FF}	0.01	0.00	0.00	0.00	0.03	0.00	0.00	0.00	0.00	0.00	0.00	0.00	0.00	0.00	0.00	0.00
t_6 - Δ^m_{DD+DA}	0.00	0.00	0.00	0.00	0.00	0.00	0.00	0.00	0.00	0.00	0.00	0.00	0.00	0.00	0.00	0.00
t_7 - Δ^m_{FF+FA}	0.00	0.00	0.00	0.00	0.00	0.00	0.00	0.00	0.00	0.00	0.00	0.00	0.00	0.00	0.00	0.00
t_8 - Δ^m_{DA+FA}	0.00	0.00	0.01	0.01	0.00	0.00	0.01	0.01	0.00	0.00	0.00	0.00	0.01	0.01	0.00	0.00
t_9 - $\Delta^m_{DD+DA+FA+FF}$	0.00	0.00	0.01	0.00	0.04	0.01	0.03	0.00	0.13	0.00	0.00	0.00	0.00	0.00	0.00	0.00
t_{10} - Δ^m_{DD-FF}	0.01	0.00	0.00	0.01	0.00	0.00	0.00	0.00	0.00	0.03	0.00	0.00	0.00	0.00	0.00	0.00
t_{11} - Δ^m_{DD-DA}	0.02	0.00	0.00	0.00	0.00	0.00	0.00	0.00	0.00	0.00	0.02	0.00	0.00	0.00	0.00	0.01
t_{12} - Δ^m_{FF-FA}	0.00	0.00	0.01	0.00	0.00	0.00	0.00	0.00	0.00	0.00	0.00	0.03	0.00	0.00	0.00	0.00
t_{13} - Δ^m_{DA-FA}	0.00	0.00	0.00	0.00	0.00	0.00	0.00	0.00	0.00	0.00	0.00	0.00	0.01	0.00	0.00	0.00
t_{14} - $\Delta^m_{(DD+DA)-(FF+FA)}$	0.00	0.00	0.02	0.03	0.00	0.00	0.05	0.01	0.00	0.04	0.00	0.00	0.02	0.10	0.00	0.00
t_{15} - $\Delta^m_{(DD+FF)-(DA+FA)}$	0.00	0.00	0.00	0.01	0.03	0.00	0.01	0.02	0.01	0.00	0.01	0.02	0.00	0.00	0.07	0.00
t_{16} - $\Delta^m_{(DD-DA)-(FF-FA)}$	0.02	0.00	0.00	0.00	0.00	0.00	0.00	0.00	0.00	0.04	0.02	0.01	0.01	0.00	0.00	0.13

Table F. Power of strategy $\mathbf{M_3}$ when applied with a nominal FDR level fixed at 20%. The power is computed as the proportions of detected QTLs (averaged over the 100 genetic architectures with $h^2 = 0.5$), displayed for each of the sixteen tests in columns. The 16 tests are numbered as $\{t_1, ..., t_{16}\}$ and each of them matches one of the QTL configurations indicated in lines (e.g. t_1 corresponds to testing $\Delta^m_{DD} = 0$, but also to the QTL configuration Δ^m_{DD})

QTL configuration \Test	t_1	t_2	t_3	t_4	t_5	t_6	t_7	t_8	t_9	t_{10}	t_{11}	t_{12}	t_{13}	t_{14}	t_{15}	t_{16}
t_1 - Δ^m_{DD}	0.37	0.00	0.00	0.02	0.12	0.03	0.00	0.00	0.01	0.08	0.06	0.02	0.01	0.00	0.05	0.00
t_2 - Δ^m_{DA}	0.04	0.03	0.00	0.01	0.00	0.02	0.00	0.00	0.02	0.02	0.01	0.00	0.02	0.01	0.01	0.00
t_3 - Δ^m_{FA}	0.00	0.01	0.02	0.00	0.01	0.01	0.01	0.01	0.00	0.00	0.01	0.02	0.01	0.00	0.02	0.01
t_4 - Δ^m_{FF}	0.01	0.00	0.01	0.22	0.06	0.02	0.04	0.01	0.01	0.07	0.00	0.04	0.00	0.01	0.00	0.02
t_5 - Δ^m_{DD+FF}	0.24	0.02	0.00	0.21	0.65	0.04	0.01	0.01	0.08	0.04	0.03	0.02	0.01	0.02	0.07	0.01
t_6 - Δ^m_{DD+DA}	0.22	0.14	0.01	0.00	0.10	0.45	0.02	0.07	0.17	0.07	0.01	0.01	0.05	0.18	0.02	0.01
t_7 - Δ^m_{FF+FA}	0.03	0.02	0.07	0.26	0.07	0.01	0.33	0.03	0.11	0.03	0.01	0.00	0.01	0.04	0.01	0.00
t_8 - Δ^m_{DA+FA}	0.01	0.09	0.03	0.01	0.00	0.02	0.04	0.16	0.09	0.05	0.06	0.00	0.02	0.02	0.11	0.01
t_9 - $\Delta^m_{DD+DA+FA+FF}$	0.25	0.10	0.07	0.32	0.66	0.43	0.40	0.16	0.80	0.03	0.00	0.00	0.02	0.09	0.00	0.01
t_{10} - Δ^m_{DD-FF}	0.28	0.00	0.02	0.37	0.05	0.03	0.00	0.01	0.01	0.72	0.04	0.06	0.02	0.12	0.00	0.17
t_{11} - Δ^m_{DD-DA}	0.26	0.05	0.00	0.00	0.11	0.01	0.00	0.03	0.02	0.12	0.42	0.00	0.04	0.00	0.17	0.16
t_{12} - Δ^m_{FF-FA}	0.01	0.00	0.12	0.25	0.05	0.00	0.01	0.02	0.00	0.04	0.00	0.42	0.05	0.00	0.13	0.13
t_{13} - Δ^m_{DA-FA}	0.00	0.04	0.05	0.01	0.00	0.05	0.02	0.02	0.02	0.00	0.03	0.03	0.28	0.11	0.00	0.14
t_{14} - $\Delta^m_{(DD+DA)-(FF+FA)}$	0.24	0.06	0.07	0.29	0.05	0.41	0.40	0.03	0.08	0.63	0.00	0.01	0.24	0.81	0.00	0.00
t_{15} - $\Delta^m_{(DD+FF)-(DA+FA)}$	0.25	0.12	0.05	0.26	0.70	0.00	0.00	0.25	0.00	0.01	0.52	0.40	0.04	0.00	0.88	0.08
t_{16} - $\Delta^m_{(DD-DA)-(FF-FA)}$	0.26	0.08	0.06	0.34	0.02	0.02	0.00	0.01	0.00	0.75	0.46	0.50	0.20	0.00	0.11	0.87

Table G. Power of strategy $\mathbf{M_3}$ when applied with a nominal FDR level fixed at 20%. The power is computed as the proportions of detected QTLs (averaged over the 100 genetic architectures with $h^2 = 0.8$), displayed for each of the sixteen tests in columns. The 16 tests are numbered as $\{t_1, ..., t_{16}\}$ and each of them matches one of the QTL configurations indicated in lines (e.g. t_1 corresponds to testing $\Delta^m_{DD} = 0$, but also to the QTL configuration Δ^m_{DD})

QTL configuration \Test	t_1	t_2	t_3	t_4	t_5	t_6	t_7	t_8	t_9	t_{10}	t_{11}	t_{12}	t_{13}	t_{14}	t_{15}	t_{16}
t_1 - Δ^m_{DD}	0.68	0.01	0.01	0.03	0.40	0.12	0.01	0.00	0.05	0.31	0.17	0.02	0.02	0.02	0.06	0.06
t_2 - Δ^m_{DA}	0.04	0.27	0.00	0.02	0.03	0.13	0.00	0.05	0.06	0.05	0.11	0.03	0.05	0.06	0.05	0.05
t_3 - Δ^m_{FA}	0.03	0.02	0.23	0.04	0.05	0.01	0.12	0.10	0.04	0.05	0.04	0.13	0.07	0.07	0.09	0.06
t_4 - Δ^m_{FF}	0.06	0.05	0.03	0.78	0.26	0.06	0.11	0.00	0.07	0.35	0.01	0.14	0.02	0.07	0.05	0.07
t_5 - Δ^m_{DD+FF}	0.74	0.01	0.01	0.70	0.99	0.13	0.11	0.00	0.36	0.16	0.12	0.14	0.02	0.09	0.42	0.03
t_6 - Δ^m_{DD+DA}	0.70	0.36	0.01	0.02	0.32	0.87	0.01	0.13	0.44	0.42	0.02	0.01	0.14	0.50	0.01	0.03
t_7 - Δ^m_{FA+FF}	0.02	0.02	0.26	0.74	0.33	0.01	0.80	0.03	0.46	0.30	0.00	0.01	0.12	0.41	0.02	0.02
t_8 - Δ^m_{DA+FA}	0.03	0.32	0.22	0.03	0.01	0.19	0.11	0.57	0.40	0.05	0.14	0.08	0.13	0.07	0.39	0.05
t_9 - $\Delta^m_{DD+DA+FA+FF}$	0.78	0.29	0.33	0.78	0.96	0.83	0.85	0.65	0.99	0.17	0.01	0.00	0.06	0.36	0.02	0.02
t_{10} - Δ^m_{DD-FF}	0.82	0.01	0.01	0.75	0.19	0.19	0.13	0.02	0.10	0.97	0.10	0.19	0.04	0.46	0.05	0.49
t_{11} - Δ^m_{DD-DA}	0.76	0.25	0.02	0.02	0.40	0.02	0.02	0.07	0.03	0.41	0.82	0.01	0.10	0.04	0.49	0.56
t_{12} - Δ^m_{FA-FF}	0.03	0.01	0.34	0.78	0.29	0.01	0.01	0.11	0.02	0.39	0.02	0.85	0.14	0.03	0.48	0.59
t_{13} - Δ^m_{DA-FA}	0.00	0.31	0.24	0.03	0.02	0.25	0.17	0.11	0.07	0.03	0.16	0.16	0.67	0.50	0.05	0.43
t_{14} - $\Delta^m_{(DD+DA)-(FA+FF)}$	0.80	0.33	0.24	0.75	0.18	0.80	0.80	0.12	0.28	0.97	0.05	0.04	0.66	0.98	0.04	0.05
t_{15} - $\Delta^m_{(DD+FF)-(DA+FA)}$	0.77	0.37	0.31	0.69	0.96	0.03	0.00	0.62	0.01	0.20	0.84	0.83	0.11	0.03	0.99	0.30
t_{16} - $\Delta^m_{(DD-DA)-(FF-FA)}$	0.69	0.29	0.32	0.77	0.25	0.02	0.04	0.08	0.03	0.97	0.78	0.87	0.72	0.02	0.29	1.00

Figures

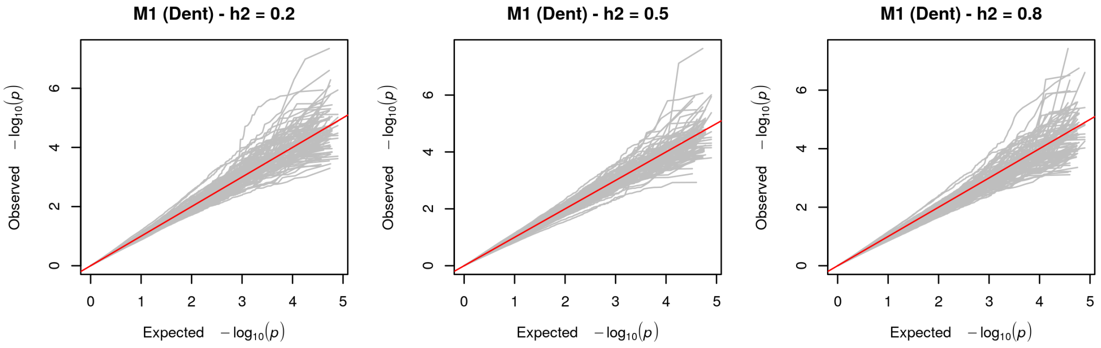

Fig A. QQ-plots of the pvalues obtained with $\mathbf{M_1}$ (Dent) applied to the chromosome H_0 for the 100 genetic architectures and 3 levels of heritability

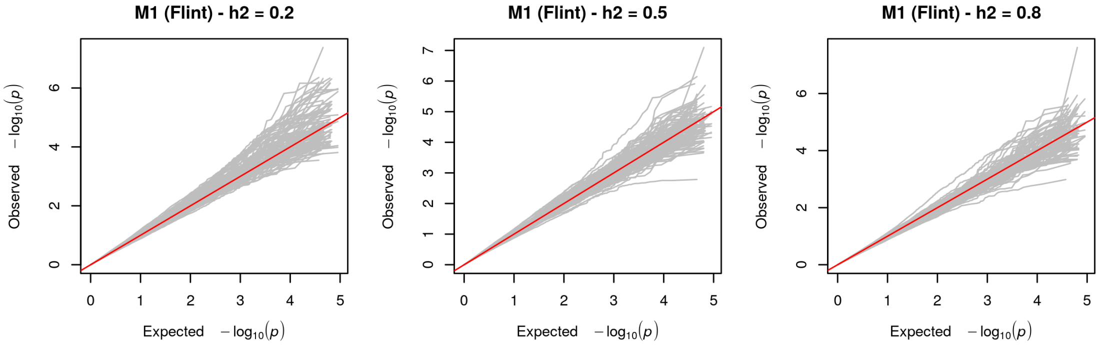

Fig B. QQ-plots of the pvalues obtained with $\mathbf{M_1}$ (Flint) applied to the chromosome H_0 for the 100 genetic architectures and 3 levels of heritability

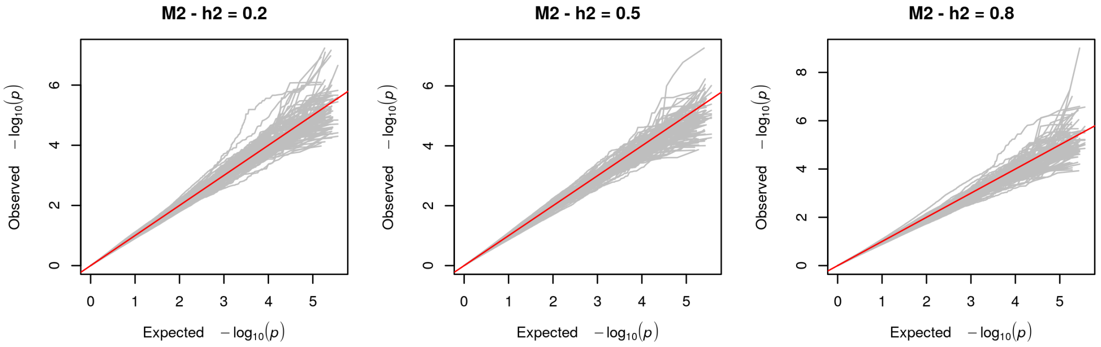

Fig C. QQ-plots of the pvalues obtained with $\mathbf{M_2}$ (globally over the four tests) applied to the chromosome H_0 for the 100 genetic architectures and 3 levels of heritability

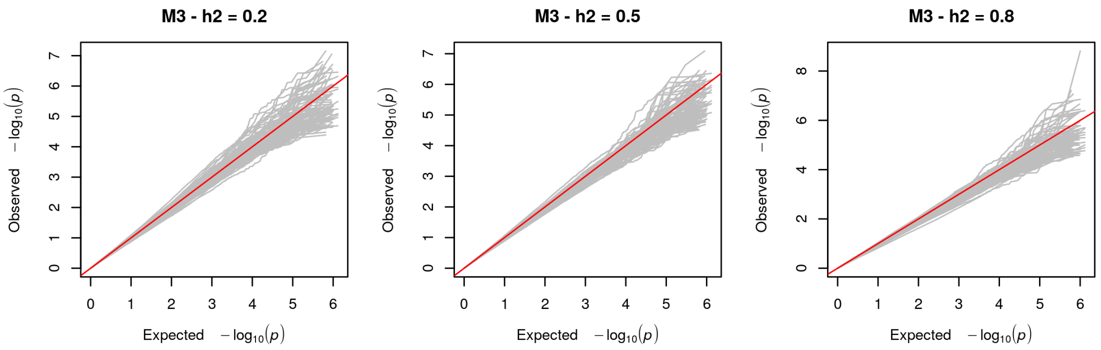

Fig D. QQ-plots of the pvalues obtained with $\mathbf{M_3}$ (globally over the sixteen tests) applied to the chromosome H_0 for the 100 genetic architectures and 3 levels of heritability

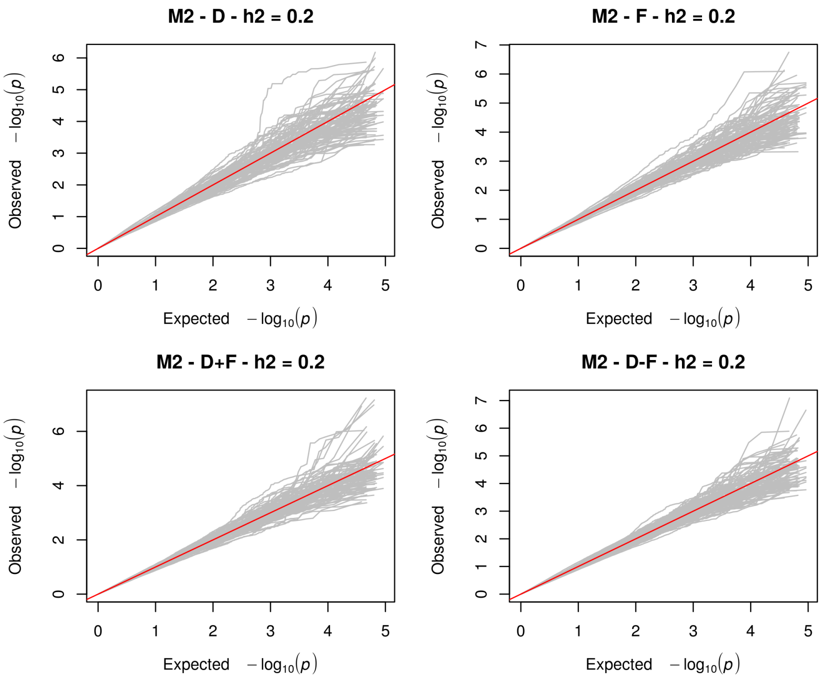

Fig E. QQ-plots of the pvalues obtained with $\mathbf{M_2}$ (separately for each test) applied to the chromosome H_0 for the 100 genetic architectures with $h^2 = 0.2$

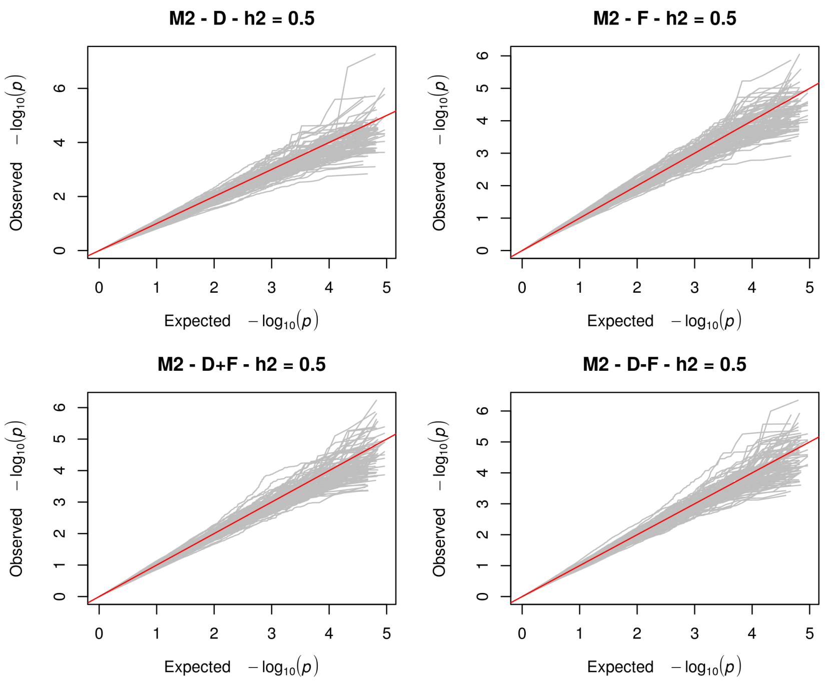

Fig F. QQ-plots of the pvalues obtained with $\mathbf{M_2}$ (separately for each test) applied to the chromosome H_0 for the 100 genetic architectures with $h^2 = 0.5$

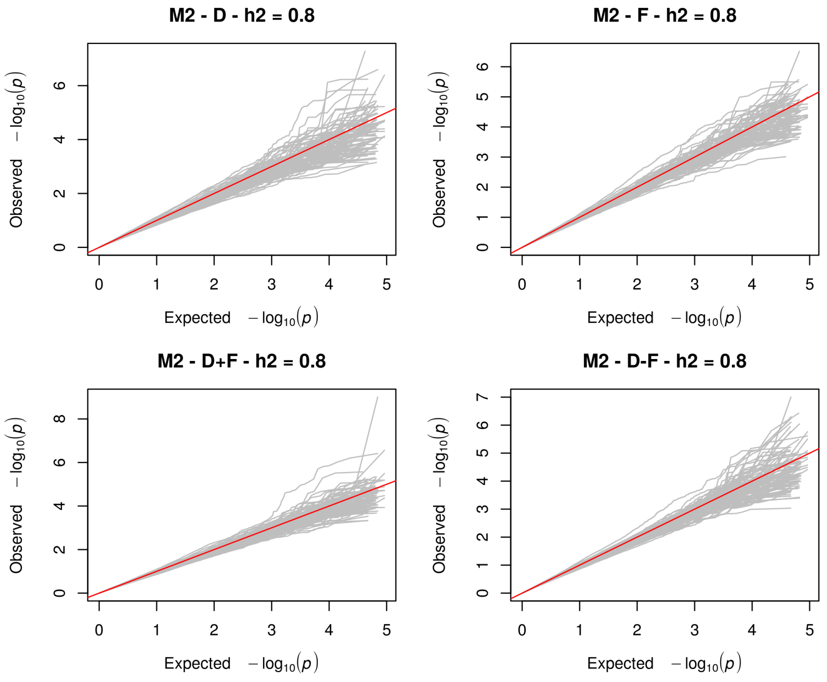

Fig G. QQ-plots of the pvalues obtained with $\mathbf{M_2}$ (separately for each test) applied to the chromosome H_0 for the 100 genetic architectures with $h^2 = 0.8$

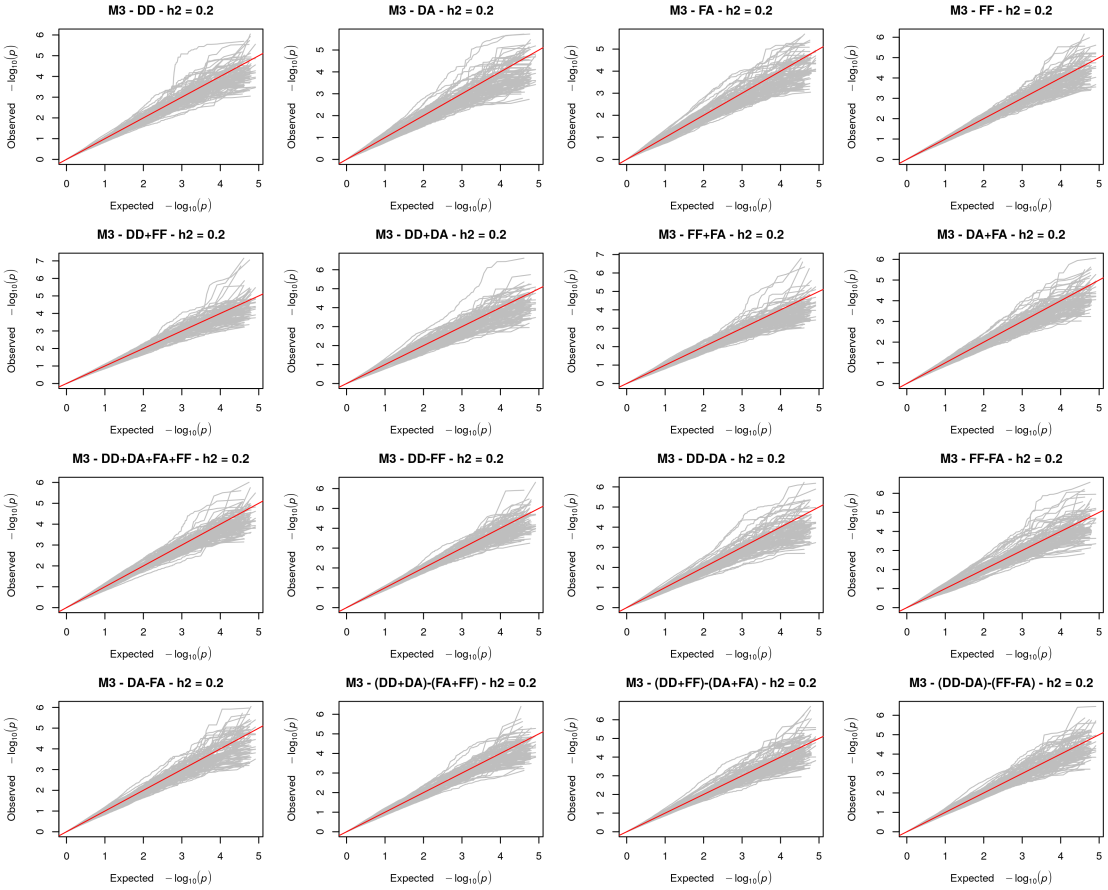

Fig H. QQ-plots of the pvalues obtained with $\mathbf{M_3}$ (separately for each test) applied to the chromosome H_0 for the 100 genetic architectures with $h^2 = 0.2$

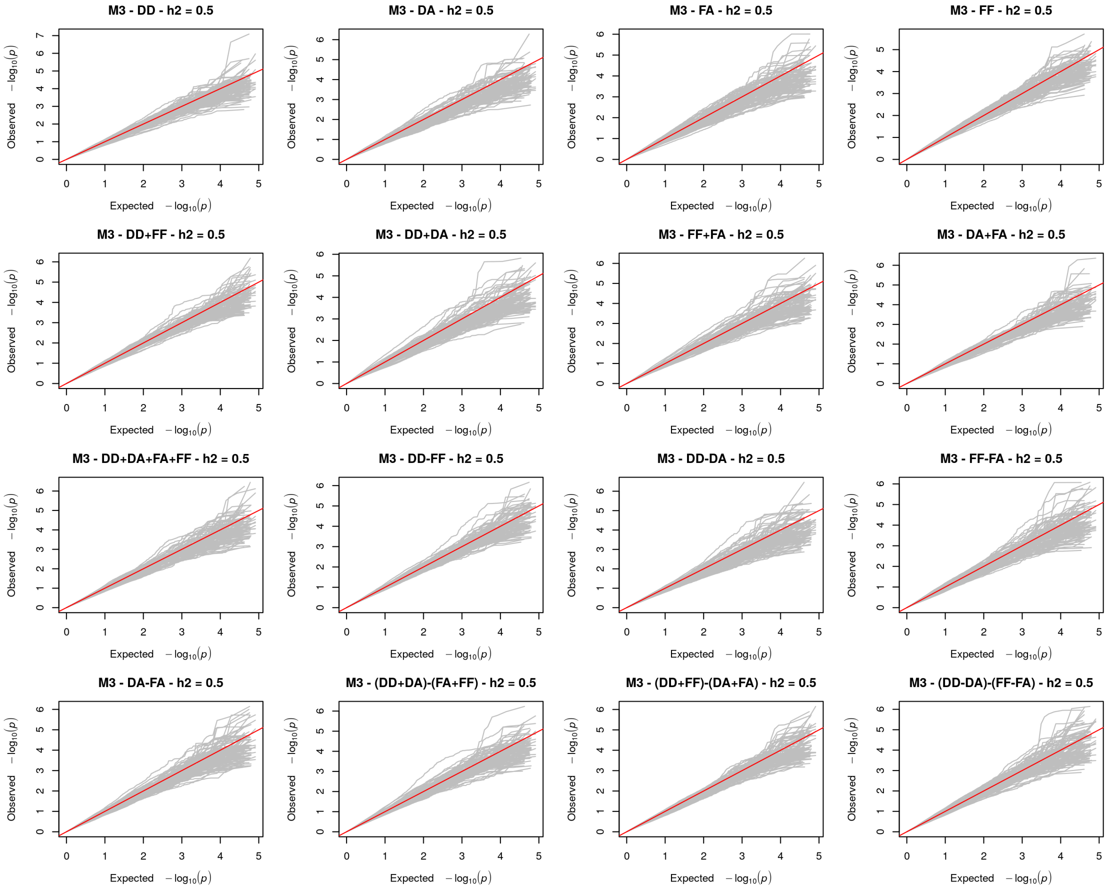

Fig I. QQ-plots of the pvalues obtained with $\mathbf{M_3}$ (separately for each test) applied to the chromosome H_0 for the 100 genetic architectures with $h^2 = 0.5$

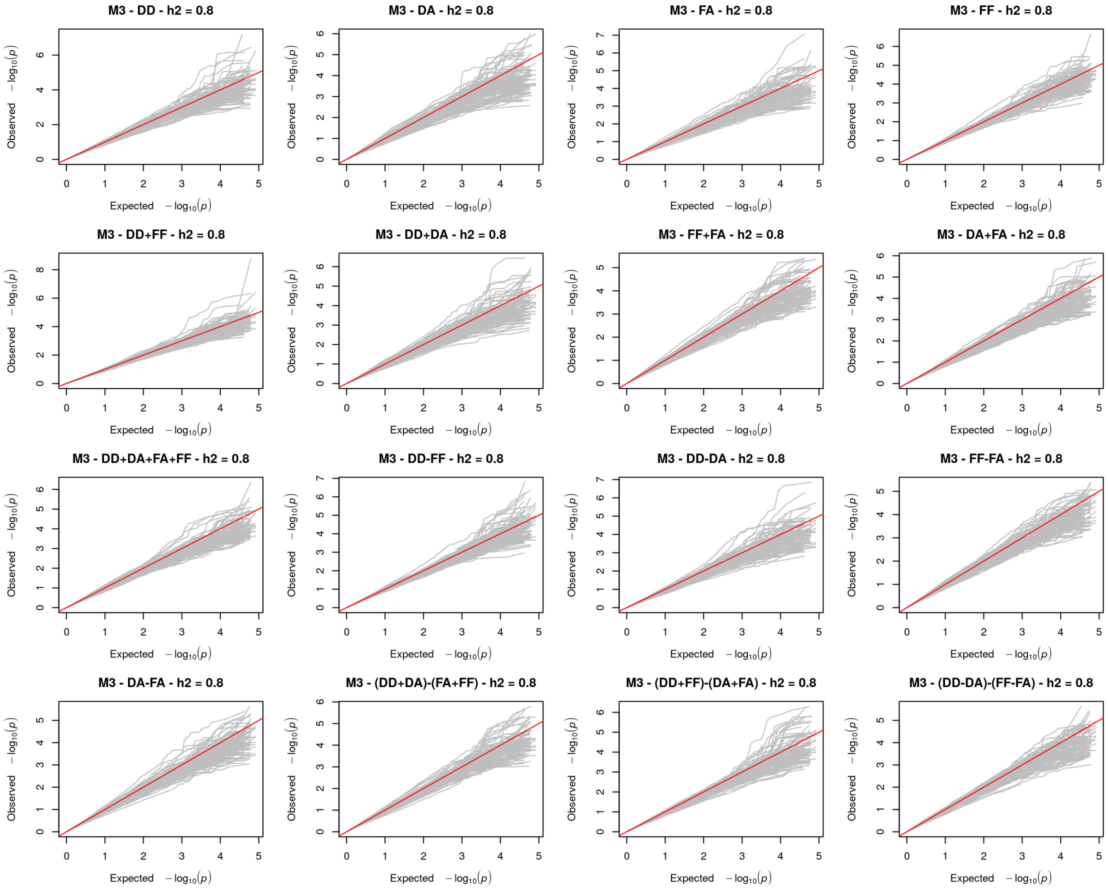

Fig J. QQ-plots of the pvalues obtained with $\mathbf{M_3}$ (separately for each test) applied to the chromosome H_0 for the 100 genetic architectures with $h^2 = 0.8$
